# 

**Published:** 1905-08

**Authors:** 


### Contributors to Vol. LXI.

AUGUST, 1905—JULY, 1906.

---

Barrows, Franklin W. . . . . . . . . . . . . . . . . . . . . . . . . . . . . . Buffalo
Beals, Lynn S. . . . . . . . . . . . . . . . . . . . . . . . . . . . . . . . . Buffalo
Breuer, Max C. . . . . . . . . . . . . . . . . . . . . . . . . . . . . . . . . Buffalo
Burke, Joseph . . . . . . . . . . . . . . . . . . . . . . . . . . . . . . . . . Buffalo
Burrows. Lorenzo, Jr. . . . . . . . . . . . . . . . . . . . . . . . . . . . . Buffalo
Coss, E. M. . . . . . . . . . . . . . . . . . . . . . . . . . . . . . . . . Cattaraugus
Cowell, Walter A. . . . . . . . . . . . . . . . . . . . . . . . . . . . . . . Olean
Cott, George F. . . . . . . . . . . . . . . . . . . . . . . . . . . . . . . . Buffalo
Crile, George W. . . . . . . . . . . . . . . . . . . . . . . . . . . . . . Cleveland
Dowd, J. Henry . . . . . . . . . . . . . . . . . . . . . . . . . . . . . . . . Buffalo
Drueck, Charles J. . . . . . . . . . . . . . . . . . . . . . . . . . . . . . Chicago
Dunham, Sidney A. . . . . . . . . . . . . . . . . . . . . . . . . . . . . . . Buffalo
Ford, Willis E. . . . . . . . . . . . . . . . . . . . . . . . . . . . . . . . . Utica
Fronczak, Francis E. . . . . . . . . . . . . . . . . . . . . . . . . . . . . Buffalo
Frye, Maud, J. . . . . . . . . . . . . . . . . . . . . . . . . . . . . . . . . Buffalo
Gram, Franklin C. . . . . . . . . . . . . . . . . . . . . . . . . . . . . . . Buffalo
Greenleaf, C. A. . . . . . . . . . . . . . . . . . . . . . . . . . . . . . Rochester
Hanley, L. G. . . . . . . . . . . . . . . . . . . . . . . . . . . . . . . . . Buffalo
Hayd, Herman E. . . . . . . . . . . . . . . . . . . . . . . . . . . . . . . . Buffalo
Howard, William Lee . . . . . . . . . . . . . . . . . . . . . . . . . . . Baltimore
Hubbell, A. A. . . . . . . . . . . . . . . . . . . . . . . . . . . . . . . . . Buffalo
Hurd, Arthur W. . . . . . . . . . . . . . . . . . . . . . . . . . . . . . . . Buffalo
Jack, George N. . . . . . . . . . . . . . . . . . . . . . . . . . . . . . . . Buffalo
Jones, Allen A. . . . . . . . . . . . . . . . . . . . . . . . . . . . . . . . Buffalo
Koyle, Frank H. . . . . . . . . . . . . . . . . . . . . . . . . . . . . . . . Hornell
Krauss, William C. . . . . . . . . . . . . . . . . . . . . . . . . . . . . . Buffalo
LeBreton, Prescott . . . . . . . . . . . . . . . . . . . . . . . . . . . . . Buffalo
Lydston, G. Frank . . . . . . . . . . . . . . . . . . . . . . . . . . . . . . Chicago
McGuire, Edgar R. . . . . . . . . . . . . . . . . . . . . . . . . . . . . . . Buffalo
McGuire, Francis W. . . . . . . . . . . . . . . . . . . . . . . . . . . . . . Buffalo
McKee, E. S. . . . . . . . . . . . . . . . . . . . . . . . . . . . . . . . Cincinnati

Meisenbach, Roland O. . . . . . . . . . Buffalo
Miller, Aaron B. . . . . . . . . . Syracuse
Miller, John A. . . . . . . . . . Buffalo
Mooney, James J. . . . . . . . . . Buffalo
Murray, Dwight H. . . . . . . . . . Syracuse
O'Gorman, Francis M. . . . . . . . . . Buffalo
Park, Roswell . . . . . . . . . . Buffalo
Potter, William Warren . . . . . . . . . . Buffalo
Price, Joseph . . . . . . . . . . Philadelphia
Price, N. Gordon . . . . . . . . . . Newark
Putnam, James Wright . . . . . . . . . . Buffalo
Rafter, John A. . . . . . . . . . North Tonawanda
Ricketts, B. Merrill . . . . . . . . . . Cincinnati
Rochester, DeLancey . . . . . . . . . . Buffalo
Seaman, Louis L. . . . . . . . . . New York
Sheehan, R. F. . . . . . . . . . Buffalo
Snell, Albert C. . . . . . . . . . Rochester
Spooner, Henry G. . . . . . . . . . New York
Stockton, Charles G. . . . . . . . . . Buffalo
Tartaro, Guiseppe . . . . . . . . . . Buffalo
Thomas, Luther A. . . . . . . . . . Buffalo
Ullman, Julius . . . . . . . . . . Buffalo
Vanduyn, E. S. . . . . . . . . . Syracuse
Van Peyma, P. W. . . . . . . . . . Buffalo
Wende, Grover W. . . . . . . . . . Buffalo
Wilson, Nelson W. . . . . . . . . . Buffalo
Young, A. A. . . . . . . . . . Newark, N. Y.

Buffalo Academy of Medicine.
Medical Association of Central New York
Medical Society of the County of Erie.
Medical Society of the County of Genesee.
Medical Society of the County of Niagara.
Niagara Falls Academy of Medicine.
Rochester Academy of Medicine

### BOOKS RECEIVED.

Treatise on Orthopedic Surgery. By Edward H. Bradford, M.D., Professor of Orthopedic Surgery, Harvard Medical School. And Robert W. Lovett, M.D., Assistant in Orthopedic Surgery, Harvard Medical School. Third edition. Octavo, pp. vi.-669. Illustrated by 592 engravings. New York: William Wood & Co. 1905. (Price: cloth, $5.00; sheep, $5.75, net prices.)

Modern Clinical Medicine. Edited by J. C. Wilson, M.D., Professor of Medicine in the Jefferson Medical College, Philadelphia. Vol. I., Infectious Diseases. Translated from Die Deutsche Klinik, under editorial supervision of Julius L. Salinger, M.D. Octavo, pp. xiv.-925. With 60 illustrations and 2 colored plates. New York and London: D. Appleton & Co. 1905. (Price, $6.00.)

A Manual of Midwifery. For Students and Practitioners. By Henry Jellett, B.A., M.D., F.R.C.P.L., Gynecologist and Obstetric Physician to Dr. Steevens's Hospital, Dublin. Small octavo, pp. xxv.-1158. 467 illustrations with 9 plates. New York: William Wood & Co. 1905. (Price: muslin, $5.50; sheep, $6.25 net prices.)

Report of the Commissioner of Education. For the year 1903. Vol. 2. Washington: Government Printing Office. 1905.

Tumors of the Cerebellum. A Symposium by Charles K. Mills, M.D., and others. New York: A. R. Elliott Publishing Co. 1905.

The Diagnosis of Diseases of Women. For Students and Practitioners. By Palmer Findley, B.S., M.D., Assistant Professor of Obstetrics and Gynecology, Rush Medical College; Assistant Attending Gynecologist to the Presbyterian Hospital, Chicago. Octavo, 588 pages, illustrated with 222 engravings and 59 plates in color and monochrome. Philadelphia and New York: Lea Brothers & Co. 1905. (Price: cloth, $4.75; leather, $5.75 net prices.)

A Textbook on the Practice of Gynecology. For Practitioners and Students. By W. Easterly Ashton, M.D., LL.D., Professor of Gynecology in the Medico-Chirurgical College of Philadelphia. Octavo, 1079 pages, containing 1046 new and original line drawings. Philadelphia and London. W. B. Saunders & Co. 1905. (Cloth, $6.50 net; half morocco, $7.50 net.)

Malaria, Influenza, and Dengue. By Dr. J. Mannaberg, Vienna, and Dr. O. Leichtenstern, Cologne. Edited, with additions, by Ronald Ross, F.R.C.S., F.R.S., Professor of Tropical Medicine, University of Liverpool; J. W. W. Stephens, M.D., D.P.H., Walter Myers Lecturer in Tropical Medicine, University of Liverpool; and Albert S. Grünbaum, F.R.C.P., Professor of Experimental Medicine, University of Liverpool. Octavo, 769 pages. Illustrated, including eight full-page plates. Philadelphia and London: W. B. Saunders & Co. 1905. (Cloth, $5.00 net; half morocco, $6.00 net.)

A Manual of Practical Hygiene. For Students, Physicians, and Medical Officers. By Charles Harrington, M.D., Assistant Professor of Hygiene in Harvard University Medical School, Boston. Third edition, revised. Octavo, 793 pages, with 118 engravings and 12 plates. Philadelphia and New York: Lea Brothers & Co. 1905. (Cloth, $4.25.)

Atlas and Textbook of Topographic and Applied Anatomy. By Professor O. Schultze, Würzburg. Edited with additions, by George D. Stewart, M.D., Professor of Anatomy and Clinical Surgery, University and Bellevue Hospital Medical College, New York. Quarto, 187 pages, containing 22 colored lithographic plates, and 89 text-cuts, 60 in colors. Philadelphia and London: W. B. Saunders & Co. 1905. (Cloth, $5.50 net.)

Textbook on the Practice of Medicine. By James M. French, M.D., Formerly Lecturer on the Theory and Practice of Medicine, Medical College of Ohio. Second revised edition. Octavo, pp. 799. Illustrated. New York: William Wood & Co. 1905. (Price, $4.00.)

Psychiatry. A Textbook for Students and Practitioners. By Stewart Paton, M.D., Associate in Psychiatry, the John Hopkins University, Baltimore, Director of the Laboratory, The Sheppard and Enoch Pratt Hospital, Towson, Md. Octavo, pp. 618. Illustrated. Philadelphia and London: J. B. Lippincott Co. 1905.

Studies from the Department of Pathology, Cornell University Medical College. Vol. IV. 1904.

Handbook of Anatomy. A Complete Compend of Anatomy, including the Anatomy of the Viscera and Numerous Tables. By James K. Young, M.D., Professor of Orthopedic Surgery, Philadelphia Polyclinic. Second edition, revised and enlarged. With 171 engravings, some in colors. Crown octavo, 404 pages. Philadelphia: F. A. Davis Company. 1905. (Flexible cloth, rounded corners, $1.50 net.)

The Surgical Assistant, A Manual for Students, Practitioners, Hospital Internes and Nurses. By Walter M. Brickner, B.S., M.D., Assistant Surgeon, Mount Sinai Hospital, Out-Patient Department. Three hundred and sixty pages, 123 original illustrations and 116 illustrations of surgical instruments. New York: The International Journal of Surgery Co. 1905. (Price, $2.00 net.)

The Treatment of Fractures; with Notes upon a Few Common Dislocations. By Charles L. Scudder, M.D., Surgeon to the Massachusetts General Hospital. Fifth edition, revised and enlarged. Octavo, 563 pages, with 739 original illustrations. Philadelphia and London: W. B. Saunders & Co. 1905. (Polished buckram, $5.00 net; half-morocco, $6.00 net.)

American Edition of Nothnagel's Practice. Diseases of the Kidney, of the Spleen, and Hemorrhagic Diseases. By Drs. H. Senator and M. Litten, Berlin. Edited with additions, by James B. Herrick, M.D., Professor of Medicine in Rush Medical College, Chicago. Octavo, 816 pages, illustrated. Philadelphia and London: W. B. Saunders & Co. 1905. (Cloth, $5.00 net; half-morocco, $6.00 net.)

Clinical Treatise on the Pathology and Therapy of Disorders of Metabolism and Nutrition. By Prof. Dr. Carl von Noorden, Physician-in-Chief to the City Hospital, Frankfurt a. M. Authorised American edition, translated under the direction of Boardman Reed, M.D., Professor of Diseases of the Gastrointestinal Tract, Hygiene and Climatology, Department of Medicine, Temple College, Philadelphia. Part VI., Drink Restriction, particularly in Obesity. New York: E. B. Treat & Co. 1905. (Price, 75 cents.)

A Manual of Acute Poisoning. With Methods for Use in First Aid to the Injured. By John W. Wainwright, M.D. New York: E. R. Pelton. 1905. (Price, 75 cents.)

A Practical Treatise on Fractures and Dislocations. By Lewis A. Stimson, B.A., M.D., Professor of Surgery in Cornell University Medical College, New York; Surgeon to the New York and Hudson Street Hospitals. Fourth edition, thoroughly revised. Octavo, 844 pages with 331 engravings and 46 plates. Philadelphia and New York: Lea Brothers & Co. 1905. (Cloth, $5.00; leather, $6.00; half morocco, $6.50, net prices.)

Saunders's Pocket Medical Formulary. By William M. Powell, M.D., author of "Essentials of Diseases of Children." Containing 1831 formulas from the best known authorities. With an Appendix containing Posological Table, Formulas and Doses for Hypodermic Medication, Poisons and their Antidotes, etc., etc. Seventh edition, revised. Philadelphia and London: W. B. Saunders & Co. 1905. (In flexible morocco, with side index, wallet, and flap, $1.75 net.)

Lea's Series of Medical Epitomes. Edited by Victor C. Pedersen, M.D. Clinical Diagnosis and Uranalysis. A Manual for Students and Practitioners. By James R. Arneill, A.B., M.D., Professor of Medicine and Clinical Medicine in the University of Colorado, Denver. Duodecimo, 244 pages, with 79 engravings and a colored plate. Philadelphia and New York: Lea Brothers & Co. 1905. ($1.00 net.)

